# There was collusion: Microbes in inflammatory bowel disease

**DOI:** 10.1371/journal.ppat.1007215

**Published:** 2018-09-20

**Authors:** Serre-Yu Wong, Ken Cadwell

**Affiliations:** 1 Kimmel Center for Biology and Medicine at the Skirball Institute, New York University School of Medicine, New York, New York, United States of America; 2 Department of Medicine, Henry D. Janowitz Division of Gastroenterology, Susan and Leonard Feinstein Inflammatory Bowel Disease Center, Icahn School of Medicine at Mount Sinai, New York, New York, United States of America; 3 Department of Microbiology, New York University School of Medicine, New York, New York, United States of America; Mount Sinai School of Medicine, UNITED STATES

## Introduction

Inflammatory bowel disease (IBD) refers to a group of chronic inflammatory diseases that affect the gastrointestinal tract and includes ulcerative colitis (UC) and Crohn’s disease (CD). UC is restricted to the mucosa of the colon, whereas the lesions in CD are often discontinuous, occur across the entire wall of the organ (transmural), and involve any part of the gastrointestinal tract from mouth to anus. Infectious etiologies have been investigated since CD was first described in 1932. In their seminal paper, Oppenheimer, Ginzberg, and Crohn describe in detail the culture conditions they used to demonstrate that CD is neither intestinal tuberculosis nor a disease caused by bacterial pathogens [1]. Over the years, the search for infectious causes of IBD has continued and even dabbled in controversy. In 1993, before his foray into the antivaccine movement with studies claiming an association between the measles, mumps, and rubella (MMR) vaccine and autism, the gastroenterologist Andrew Wakefield published that measles virus particles could be found in tissue specimens from CD patients [2]. The results were never reproduced [3]. On the other hand, early life antibiotic usage has been associated with IBD, and somewhat paradoxically, antibiotics are sometimes used as adjunctive treatment in IBD, implicating bacteria in the disease [4]. It has now been over 80 years since CD was described, and the role of microbes in IBD remains unclear.

While a single causative agent has remained elusive, the origin of IBD may be explained by an aberrant response to the community of microbes that reside in the gut, the microbiota. Other excellent reviews have articulated how shifts in bacterial communities occur and subsequently sustain IBD. However, this role for the microbiota does not preclude the possibility that specific infectious agents contribute to IBD as key members of the microbiota with a disproportionate effect akin to a pathogen, and nonbacterial agents have not received sufficient consideration. In this pearl, we review the myriad microbes that have been interrogated in patients and laboratory models for their roles in IBD pathogenesis, highlighting the central importance of understanding host–microbe interactions.

### Infectious triggers

The existence of a pathogen trigger is intriguing because the intestinal pathology of IBD is similar to that which occurs during infection by *Mycobacterium*, *Yersinia*, *Salmonella*, *Shigella*, and *Campylobacter* species [5]. For instance, CD and intestinal tuberculosis share a predilection for the terminal ileum and the presence of granulomas by histopathology, and *Mycobacterium avium paratuberculosis* (MAP) causes Johne’s disease in ruminants, which bears a striking resemblance to CD. Multiple groups have tested thousands of IBD samples for MAP without reaching a definitive conclusion [6]. Rather than directly initiating IBD, pathogens may be responsible for exacerbating or sustaining disease. *Clostridium difficile* colonization and cytomegalovirus (CMV) reactivation are associated with IBD flares, though likely as a consequence of inflammation or medications. In addition to classical pathogens, commensal organisms with disease-causing potential, referred to as pathobionts, have received attention, including *Bacteroides fragilis*, *Helicobacter hepaticus*, and *Enterococcus faecalis*. In particular, adherent-invasive *Escherichia coli* (AIEC) isolated from ileal tissue of CD patients has received attention [7]. AIEC and other *Enterobacteriaceae* species utilize electron acceptors such as nitrate that are byproducts of the host inflammatory response, which can aggravate inflammation and cause specific disease manifestations, such as colorectal cancer [8, 9]. Targeting the respiratory pathways in pathobionts may be a reasonable intervention strategy [10].

**Fig 1:**
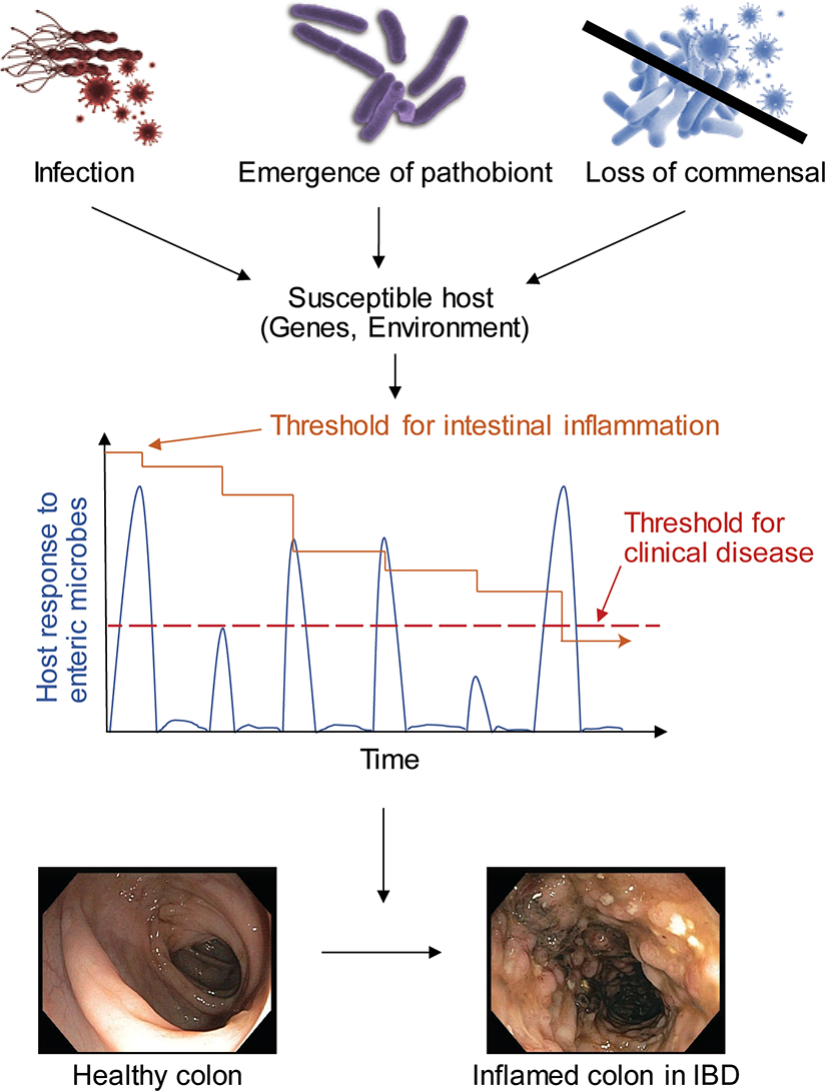
Model for the role of microbes in IBD pathogenesis. Variable changes to the microbiome, whether by an infectious agent, emergence of a pathobiont, or loss of protective commensals, affect an individual who is susceptible via genetic risk alleles or environmental factors, including diet or lifestyle such as smoking. These episodes cause host responses to the enteric microbes (blue spikes) that over time eventually immobilize host protective mechanisms such that the threshold for intestinal inflammation (orange line) declines to the point of crossing the threshold for symptomatic clinical disease (dashed red line), thereby resulting in IBD. IBD, inflammatory bowel disease.

The role of infectious agents may be to incapacitate host protective mechanisms, thereby lowering the threshold for subsequent inflammation to occur (Fig 1). Episodes of acute gastroenteritis, most commonly *Salmonella* and *Campylobacter*, precede the onset of IBD in some patients [11]. Interestingly, recurrent cycles of *Salmonella* infection and resolution in mice lead to chronic intestinal inflammation via loss of a protective mechanism of lipopolysaccharide detoxification [12]. Also, acute infection by the protozoan *Toxoplasma gondii* leads to a break in immune tolerance toward commensal bacteria by inducing the differentiation of microbiota-reactive T cells [13]. Thus, while IBD does not appear to be transmissible, intestinal microbes likely play active roles in inducing IBD.

### Gene–microbe interactions

Genome-wide association studies have implicated approximately 200 genetic loci in IBD, but even the most highly associated risk alleles confer only a modest increase in susceptibility [14]. Monogenic forms of IBD exist in which rare mutations (such as in the IL-10 receptor) lead to severe disease in early life, but incomplete penetrance and interindividual heterogeneity suggest environmental variables are important [15]. Thus, a generally accepted paradigm for IBD pathogenesis is that environmental factors and intestinal microbiota conspire to induce inflammation in genetically susceptible individuals.

Animal models support this paradigm. Mice deficient in IL-10–signaling display significant differences in disease severity and penetrance, depending on the presence of pathobionts in the mouse facility, most notably *Helicobacter* species [15]. IL-10 is an anti-inflammatory cytokine produced by regulatory T cells that blocks Th1 and Th17 cells reactive against the microbiota. Germ-free *Il-10*^*−/−*^ mice, which are otherwise resistant to colitis in the absence of the microbiota, colonized with *E*. *coli* and *E*. *faecalis* together develop bacteria-specific T-cell responses and inflammation [16]. Observations made with mice harboring mutations in nucleotide-binding oligomerization domain-containing protein 2 (*Nod2*) and *Atg16L1*, which are among the highest risk factors for CD, also implicate gene–microbe interactions. NOD2 is a cytosolic sensor of microbial ligands. In addition to displaying susceptibility to model enteric pathogens such as *Citrobacter rodentium* [17], *Nod2*-deficient mice develop CD-related pathologies in the small intestine following colonization by the pathobionts *H*. *hepaticus* or *Bacteroides vulgatus* [18, 19]. ATG16L1 mediates autophagy, a pathway that promotes homeostasis through recycling of cellular parts, including organelles or proteins. Inhibition of ATG16L1 or other autophagy proteins increases susceptibility to many pathogens but paradoxically leads to enhanced production of cytokines, including IL-1β, due to accumulation of damaged mitochondria that produce reactive oxygen species (ROS) and potentiate inflammasome activity [20]. *Atg16L1* mutant mice display structural and functional defects in antimicrobial epithelial cells in the small intestine called Paneth cells, similar to CD patients carrying the *ATG16L1* risk allele [21]. These and other intestinal pathologies in *Atg16L1* mutant mice depend on infection by murine norovirus (MNV) [22], raising the possibility of a virus–gene interaction in IBD. MNV induces necrotic death in the intestinal epithelium downstream of tumor necrosis factor α (TNFα) production [23]. On the other hand, *Atg16L1* mutant mice mount an enhanced monocyte response that confers resistance to infection by *C*. *rodentium* [24], a striking contrast to observations made in the *Nod2* mutants.

These examples illustrate how gene–microbe interactions can be highly specific. Overt inflammation in these various animal models frequently requires an additional “hit,” such as chemical injury to the epithelium or a second mutation (Fig 1). Hence, individual infectious agents, although potentially necessary, are unlikely to be sufficient to trigger IBD. We classify microbes as commensals, pathobionts, and pathogens for expediency, but other variables, including host genetics, determine whether a microbe is pathogenic.

### Beyond bacteria

MNV is a positive-strand RNA virus related to noroviruses that cause gastroenteritis in humans. Although persistent MNV infection induces CD-like pathologies in the *Atg16L1* mutant mice, the same viral strain is beneficial in a nonmutant setting. MNV can compensate for the absence of certain bacteria by promoting intestinal development, lymphocyte function, and resistance to injury [25]. Thus, MNV provides similar cues to the host as bacterial members of the microbiota and mediates a disease associated with the microbiota in a genetically susceptible host. Most, if not all, humans will be infected by enteric viruses including noroviruses, often asymptomatically and multiple times. Additionally, interactions between enteric viruses and the bacterial microbiota are pervasive and include physical interactions that facilitate attachment to the animal host cell [26]. These findings indicate that the collection of intestinal viruses, the gut virome, may function as a component of the microbiome and is a key player in IBD. Sequencing of colonic tissue revealed an enrichment of DNA viruses, such as adenovirus, in IBD patients [27]. A larger study in which viral particles in the stool were analyzed identified an increased presence of anellovirus in patients and a major enrichment of *Caudovirales* bacteriophages [28]. Bacteriophages potentially serve as provocateurs of inflammation by shaping bacterial community structure and mediating horizontal gene transfer of virulence factors.

Archaea are present in the gastrointestinal tract and produce organic acids, reaction end products, and hydrogen waste in the process of polysaccharide fermentation [29]; their functions in intestinal inflammation remain to be directly interrogated. In contrast, progress has been made in characterizing the role of fungi and protozoa in intestinal inflammation. Antibodies to *Saccharomyces cerevisiae* were found to be associated with CD patients 30 years ago [30]. More recently, *S*. *cerevisiae* colonization in mice was shown to promote colitis via enhanced purine metabolism [31]. Although the significance of antibodies against *S*. *cerevisiae* remains unclear, a role for the fungal communities in the gut (mycobiome) in IBD is supported by studies examining the innate immune receptor for fungal glucans Dectin-1 (*CLEC7A*). A *CLEC7A* polymorphism is associated with severe UC, and mice lacking Dectin-1 are susceptible to colitis [32]. While protozoa such as *Giardia lamblia* and *Entamoeba histolytica* cause diarrhea, there are also gastrointestinal protozoans that blur the line between commensal and pathogen. IL-18 produced in response to colonization by *Tritrichomonas* species induces a Th1 and Th17 response that protect against *Salmonella* Typhimurium while also exacerbating T-cell mediated colitis [33, 34]. Clearly, studies that seek to understand IBD origin need to consider nonbacterial agents.

### Gain of harmful microbes or loss of beneficial ones?

Considering the microbiome as an ecosystem, it seems evident that interactions between species contribute to intestinal homeostasis and disease. Microbes cooperate through such processes as quorum sensing and consumption of each other’s metabolic byproducts. For example, *Bacteroides ovatus* engages in polysaccharide digestion to feed *B*. *vulgatus*, which in turn increases *B*. *ovatus* fitness [35]. It follows that loss of certain species could disrupt the community structure in a manner that promotes disease (Fig 1). Some versions of the hygiene hypothesis propose that the lack of exposure to certain infectious agents (potentially during a developmental window) is responsible for the rise in inflammatory diseases that coincides with industrialization. Parasitic worms known as helminths are conspicuously absent in populations with high IBD incidence. In the *Nod2* mutant mouse model of CD mentioned above, helminth infection increases the abundance of *Clostridiales* species that inhibit colonization by the disease-causing *B*. *vulgatus* [36]. Helminth infection was also found to enrich colonization by *Clostridiales* and reduce *Bacteroidales* in humans [36], providing evidence that individuals can lose anti-inflammatory members of the microbiota under conditions associated with industrialization.

### Outlook

The following observations explain why a causative agent of IBD has eluded identification:

1. Taxonomically distinct microbes evoke similar responses from the host, which would explain why multiple agents of gastroenteritis are implicated in IBD.
2. According to the gene–microbe interactions paradigm, an infectious agent would only trigger disease in individuals harboring the cognate susceptibility variant. Genetic information may be necessary to reveal an association with an infectious agent.
3. Many of the infectious agents mentioned above cause self-resolving infections, raising the possibility that the causative agent would no longer be present at the time of a study.
4. Most clinical studies are not equipped to incorporate nonbacterial residents of the intestine in their analysis.
5. The absence of key beneficial microbes may be a stronger factor than the pathogenic microbes that replace them and induce disease.

To overcome these barriers, we advocate longitudinal analysis of potential IBD agents in a kingdom-agnostic and hypothesis-driven manner in well-defined patient cohorts. Targeted approaches must be used in parallel with metagenomics because incomplete or incorrect annotation of biological entities, biases introduced through sample processing, and suboptimal thresholds of detection remain problematic in deep-sequencing approaches. Also, species-level resolution may not be sufficient due to strain-specific virulence factors. In a remarkable example, investigators synthesized host genetic information and microbiology to identify two bacterial subspecies (Enterotoxigenic *B*. *fragilis* and polyketide synthase–positive *E*. *coli*) that act in concert to cause colorectal cancer in a subset of patients harboring a germline mutation in the Adenomatous polyposis coli (*APC*) tumor suppressor gene [37]. The localization of these two bacteria within a biofilm is significant because it indicates that key species may be missed if we sample stool instead of affected tissue. This discovery was made possible by prior studies, spanning over a decade, using clinical specimens, cell culture, and animal models. As an increasing number of institutions are establishing large tissue repositories and patient cohorts, we believe similar discoveries are possible in IBD.
